# Differential Co-Expression between α-Synuclein and IFN-γ Signaling Genes across Development and in Parkinson’s Disease

**DOI:** 10.1371/journal.pone.0115029

**Published:** 2014-12-10

**Authors:** Noa Liscovitch, Leon French

**Affiliations:** 1 The Leslie and Susan Gonda Multidisciplinary Brain Research Center, Bar Ilan University, Ramat Gan, Israel; 2 Rotman Research Institute, Baycrest Hospital, Toronto, Ontario, Canada; Hertie Institute for Clinical Brain Research and German Center for Neurodegenerative Diseases, Germany

## Abstract

Expression patterns of the alpha-synuclein gene (SNCA) were studied across anatomy, development, and disease to better characterize its role in the brain. In this postmortem study, negative spatial co-expression between SNCA and 73 interferon-γ (IFN-γ) signaling genes was observed across many brain regions. Recent animal studies have demonstrated that IFN-γ induces loss of dopamine neurons and nigrostriatal degeneration. This opposing pattern between SNCA and IFN-γ signaling genes increases with age (rho = −0.78). In contrast, a meta-analysis of four microarray experiments representing 126 substantia nigra samples reveals a switch to positive co-expression in Parkinson’s disease (p<0.005). Use of genome-wide testing demonstrates this relationship is specific to SNCA (p<0.002). This change in co-expression suggests an immunomodulatory role of SNCA that may provide insight into neurodegeneration. Genes showing similar co-expression patterns have been previously linked to Alzheimer’s (ANK1) and Parkinson’s disease (UBE2E2, PCMT1, HPRT1 and RIT2).

## Introduction

Parkinson's disease (PD) is a common neurodegenerative disorder that causes severe motor and cognitive disabilities. The majority of PD cases are sporadic, with no known cause of onset [1]. Some cases of PD have a genetic basis; in familial PD and a small percentage of the sporadic cases, the underlying cause is related to a mutation in certain genes. Common variants identified in genome wide association studies explain around 5% of cases, with 27% phenotypic variance explained by all common variants [2]. The limited explanatory power of these past findings suggests genetic and environmental risks combine in complex ways to cause PD.

We focused our study on the alpha-synuclein gene (SNCA), which provided the first causative mutation for PD [3]. SNCA protein fibrils are main components of Lewy bodies and glial cytoplasmic inclusions. These abnormal protein aggregates mark neurons and glia in brains affected by PD, Lewy body dementia and multiple system atrophy [4]. Multiplications of the SNCA gene are implicated in familial PD [4]. Several point mutations of the α-synuclein protein are associated with the development of PD [5]. Further, several common single nucleotide variants in SNCA are associated with increased susceptibility to sporadic PD. However, the direct role that SNCA plays in the progression of PD, and the relation between SNCA and PD onset are still largely unknown [6].

We first use an anatomically comprehensive atlas of normal brain tissue to provide a regional characterization of SNCA expression. In order to functionally associate SNCA expression, we examined its co-expression dynamics across brain regions and development. Interestingly, we find strong and significant negative co-expression between SNCA and genes that participate in immune responses, specifically genes in the interferon-gamma (IFN-γ) mediated signaling pathway. Negative co-expression with IFN-γ signaling genes is highly significant and robust, and is found in each of the gene expression resources used. We examined these negative correlations over development and found a strong developmental effect. Specifically, brain-wide expression patterns of IFN-γ signaling genes is increasingly opposite of SNCA over development. We tested this co-expression relationship in several gene expression datasets of PD cases, revealing a switch to positive co-expression between SNCA and IFN-γ signaling genes in diseased brains.

## Materials and Methods

### Developmental and Spatial Gene Expression

To characterize SNCA expression and co-expression in the brain across regions and development we used gene expression data from three Allen Institute sources: the Allen Human Brain Atlas [7], the Allen Prenatal Laser Microdissection (LMD) dataset [8] and BrainSpan [9]. Neuroanatomical expression data from the Human Brain Atlas was averaged across probes and brain regions for each donor to determine regional enrichment (downloaded on February 6th, 2013). We used the probe to gene mappings provided by the Allen Institute. This averaging provides donor specific gene by region expression profiles that range in size from 185 to 348 brain regions that provide expression data for 29176 genes (mix of gene symbols and probes not mapped to genes). Donor age ranges from 24 to 57 years old with one female and five male (more information available at http://human.brain-map.org/). We performed the same averaging on the Allen Prenatal Laser Microdissection (LMD) Microarray dataset that measures expression in 222 to 339 brain regions in four donors (15 to 21 postconception weeks, one male and three female brains).

Gene expression data from the BrainSpan resource was used to examine developmental expression patterns [9]. Although lower in regional resolution than the Human Brain Atlas, it provides both exon array and RNA-sequencing expression data for human brain samples covering 41 timepoints between 8 postconception weeks and 40 years of age (578 samples total). The number of sampled brain regions ranges between 2–16 for each of the 41 donors. At least 14 regions are sampled in the majority of donors. We relied primarily on the RNA sequencing (RNA-seq) data but also report results from the exon arrays. We used the gene summarized expression data which contains profiles for 22,327 genes across 488 samples (RNA-seq).

### Gene Expression Profiles of Parkinson’s Disease

We analyzed several datasets of gene expression measured in blood and brains of PD patients (Table 1). Four publicly available postmortem datasets that measured substantia nigra gene expression in at least 20 postmortem and control brains were found using literature searches [10]–[13]. A fifth study that assayed gene expression in blood of 105 subjects was also used for comparison to the brain samples [14]. PD microarray datasets were downloaded from and pre-filtered by Gemma [15]. This pre-filtering removes probes expressed at very low levels and those with a large proportion of missing values. Meta p-values were computed using the Fisher’s trend method as implemented in the MetaP software (Dongliang Ge, http://compute1.lsrc.duke.edu/softwares/MetaP/metap.php).

**Table 1:** Differential co-expression between SNCA and IFN-γ genes in Parkinson’s gene expression studies.

		Mean Correlation		Tests of Differential Co-expression			
Name	Samples	Normal	PD	Wilcoxon	Permutation	Inversions	Permutation
GSE6613 - Scherzer(Blood)	105	−0.115	−0.115	0.85	0.508	4.48%	0.974
GSE7621 - Lesnick	25	−0.166	0.099	4.30E-05	0.101	57.14%	0.037
GSE8397 - Moran	39	−0.231	−0.036	3.32E-04	0.086	32.84%	0.046
GSE20295 - Zhang	29	−0.154	−0.014	0.020	0.219	31.25%	0.115
GSE20159 - Zheng	33	−0.040	0.183	6.06E-04	0.045	34.62%	0.014
Fisher’s Trend Meta P-value (substantia nigra datasets):				1.32E-10	0.0041		0.0003

The last dataset (GSE20159 – Zheng) assayed gene expression in cases of mild PD related Lewy body neuropathology. Paired Wilcoxon and empirical permutation tests that shuffled disease labels were used to test differential co-expression. The “Inversions” column shows the proportion of IFN-γ genes that switch from negative co-expression with SNCA in normal control samples to positive in Parkinson’s cases.

### Data Preprocessing

Gene level expression profiles were ranked for all datasets, resulting in a single ranked list of genes per sample. This ranking was applied to reduce possible batch effects in the microarray datasets and cross sample inconsistencies.

### Functional analysis

To functionally characterize the genes negatively and positively co-expressed with *SNCA*, we calculated the spatial correlation of each gene in the dataset with SNCA. We ranked the genes based on the correlations in an ascending and descending order and performed a Gene Ontology (GO) enrichment analysis on ranked gene sets using GOrilla [16].

## Results

### Regional expression of SNCA in the adult human brain

**Figure 1:**
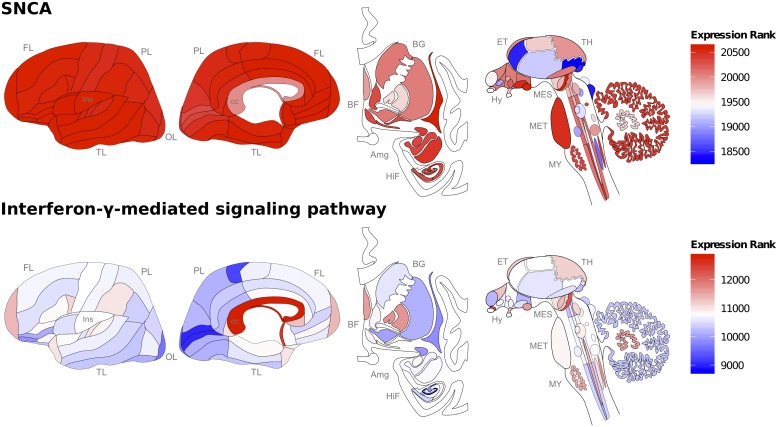
Brain map showing ranked gene expression of SNCA and IFN-γ-mediated signaling pathway genes. Expression ranges from high (red) to low (high), with the two maps showing disjoint ranges. Ranked expression was averaged across donors and subregions. Displayed expression for the interferon pathway gene set is the median across genes. Abbreviations for the brain regions: frontal lobe (FL); parietal lobe (PL); temporal lobe (TL); occipital lobe (OL); basal forebrain (BF); basal ganglia (BG); amygdala (AmG); hippocampal formation (HiF); epithalamus (EP); thalamus (TH); hypothalamus (Hy); mesencephalon (MES); metencephalon (MET) and myelencephalon (MY).

SNCA is highly expressed across the cortex, notably the claustrum, insula, and temporal lobe (Fig. 1, S1 Table). Of the 414 profiled regions in the Allen Human Brain atlas, the superior rostral gyrus has the highest SNCA expression levels but was only assayed in four donors. To increase sample size we filtered for regions that were assayed in all six brains (105 left hemisphere regions). In this reduced set of regions, the substantia nigra pars compacta has the second highest median SNCA expression. The short insular gyri and CA2 field are ranked first and third, respectively. In contrast, the substantia nigra pars reticulata has below average expression (ranked 88th of 105). Overall, this pattern agrees with past studies of SNCA expression and PD pathology [17], [18].

### SNCA and interferon-γ mediated signaling pathway genes are negatively co-expressed

We used the “guilt by association” approach to find genes with similar expression patterns to SNCA across the Allen Human Brain atlas (spatial co-expression) [19]. Functions of the resulting genes with correlated expression (co-expression) across the brain suggest valuable associations. Given the age related onset of PD we computed regional SNCA co-expression separately in the six adult brains in the atlas (ages 24–57). For each brain we used gene ontology enrichment analysis to extract functional groups associated with most extreme co-expressed genes (see Methods). For positively co-expressed genes we observed that “synaptic transmission” was consistently top ranked (S2 Table), in agreement with past studies [20]. Interestingly, genes that are most negatively correlated with SNCA are enriched for immune response associations. Example categories are “type I interferon-mediated signaling pathway”, “interferon-gamma-mediated signaling pathway” and “positive regulation of T cell mediated cytotoxicity”. Gamma or type II interferon-mediated signaling pathway (73 genes, corrected p<0.001, mean Spearman rho = −0.218) was significantly co-expressed with SNCA in 3 of the 6 donors (S3 Table); and is defined as “A series of molecular signals initiated by the binding of interferon-gamma to a receptor on the surface of a cell, and ending with regulation of a downstream cellular process” (GO: 0060333, S4 Table). In one donor (H0351.2002), it is the most significant GO category (FDR corrected p = 1.2*10^−4^).

Gamma interferon mediated signaling pathway genes (henceforth referred to as IFN-γ genes) are expressed at variable but low levels in the six brains assayed in Allen Human Brain Atlas (Fig. 1). The high spatial resolution of this atlas allowed identification of regions responsible for the negative correlation between IFN-γ genes and SNCA. Using a leave-one-region-out approach, the three top-contributing regions are the central glial substance, the hypoglossal nucleus and the spinal trigeminal nucleus. Within major brain divisions the telencephalon (rho = −0.134) and metencephalon (rho = −0.206) provide the lowest negative spatial correlations.

### Spatial correlation between SNCA and IFN-γ genes decreases with age

**Figure 2:**
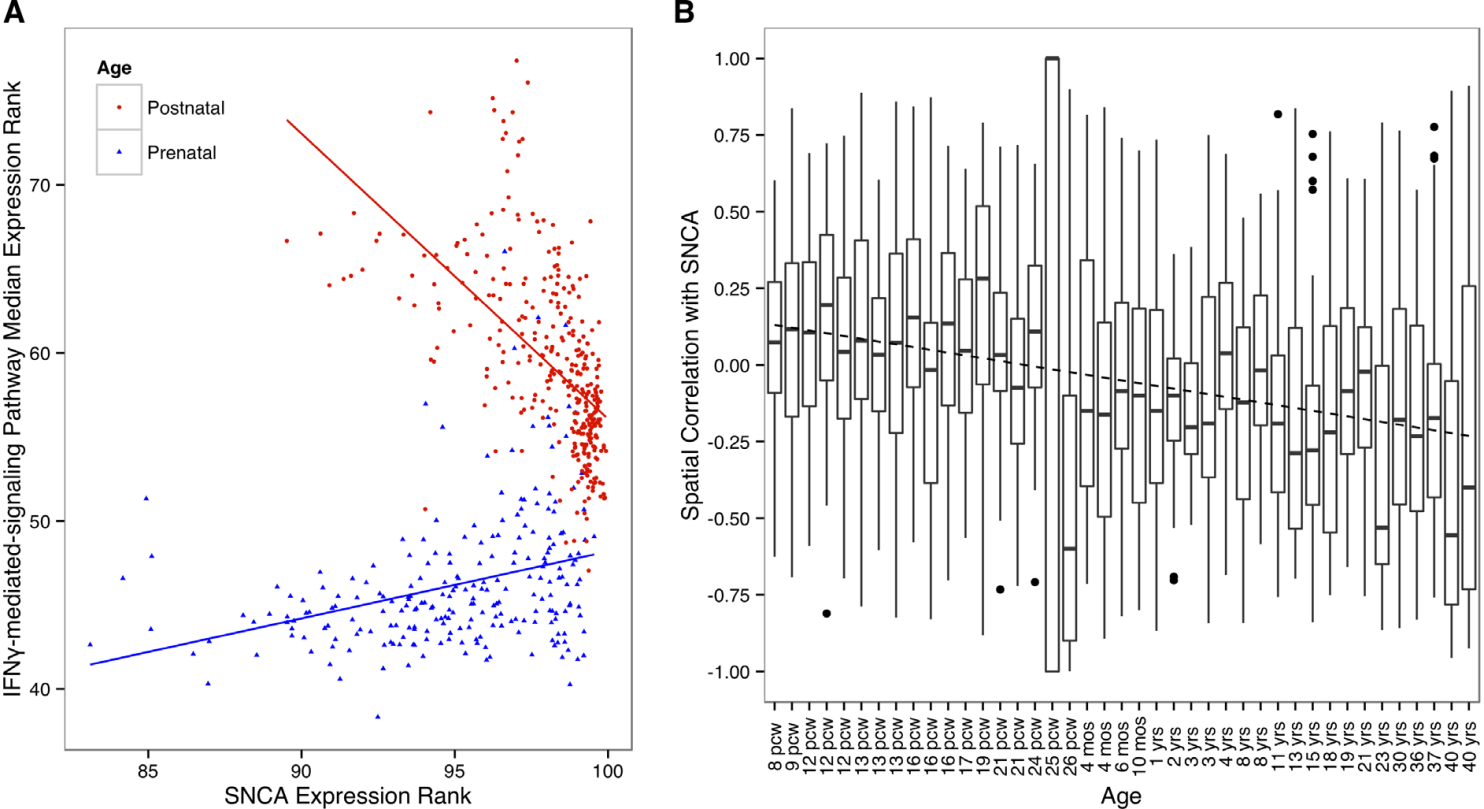
Differential co-expression between IFN-γ genes and SNCA across age. A) Scatterplot of SNCA and IFN-γ-mediated-signaling pathway median expression in prenatal (red triangles, n = 232) and postnatal samples (blue circles, n = 345). High rank corresponds to high expression. An outlier prenatal sample with low SNCA expression of 16524 was excluded to improve plotting. B) Boxplots showing spatial correlation of the 73 IFN-γ genes against SNCA within each BrainSpan donor. The 25 pcw (postconception weeks) fetus has only two sampled brain regions which results in a Spearman correlation of −1 or 1.

The previous finding of age-progressive neurodegeneration of nigrostriatal-tract neurons following over-expression of IFN-γ motivated our analysis of co-expression across age [21]. In the BrainSpan samples, expression of SNCA and IFN-γ genes show opposing brain-wide patterns as donor age increases (Fig. 2). This age associated in decrease in spatial correlation is highest between SNCA and the Suppressor of Cytokine Signaling 1 (SOCS1) (rho = −0.743, p<9.4*10^−8^). We find a stronger decrease for the whole gene set when using mean correlation between SNCA and the 73 IFN-γ genes (rho = −0.783, p = 1.4e-09). In contrast, Protein Inhibitor of Activated STAT, 1 (PIAS1) shows the highest increase in correlation with age (rho = 0.623, p<0.0001). For the SOCS1 gene the spatial correlation with SNCA drops from 0.1 in the youngest (8 post-conception weeks) to −0.61 and −0.8 in the two oldest brains (40 years old). We note that SOCS1 increases in IFN-γ activated microglia and is regulated by Parkinson protein 7 [22]. Fig. 2A demonstrates the relatively low expression of the IFN-γ genes before birth. In this coarse grouping of pre- and postnatal samples, the SNCA to IFN-γ gene group correlation drops from 0.106 in the prenatal samples to −0.191 after birth. To calculate an empirical p-value we tested 10,000 random gene sets of the same size, resulting in no sets with an age-dependent SNCA spatial correlation below −0.783 (p<0.0001). This suggests that the SNCA anti-correlation is specific to IFN-γ genes. To test specificity of SNCA we computed the same correlations with IFN-γ genes for all genes. Only 6 genes have a stronger decreasing relationship with the IFN-γ genes (HPRT1, NAP1L2, UBE2E2, MORF4L2, ZC3H15, PCMT1, p<2.7*10^−4^). We note that three of these are linked to PD. Specifically, HPRT1 mutations are known to cause Lesch-Nyhan syndrome which has Parkinsonian features and basal gangila dopamine depletion [23], [24] and PCMT1 is suspected to increase neuronal cell death in PD [25]. Thirdly, UBE2E2 is known to interact with the parkin protein [26].

The BrainSpan dataset provides exon array expression data for a subset of the samples assayed with RNASeq (508 samples). These additional expression profiles allow control for method of gene expression measurement. The same decreasing correlation between SNCA and the interferon-γ genes holds in the exon array data (rho = −0.617, S1 Figure). Empirical testing shows this is a specific result based on replacing IFN-γ genes with random sets of the same size (p = 0.006) and replacing SNCA with other genes (p = 0.0024, 41 genes).

### High-resolution spatial co-expression replicates age difference

We next tested the differential co-expression between SNCA and IFN-γ genes across the whole brain by joining the adult Allen Human Brain Atlas and with the Allen Prenatal Laser Microdissection (LMD) Microarray samples (assayed with same microarray platform). The result is expression data from six adult and four prenatal brains (15–21 post conception weeks) that have been assayed across hundreds of brain regions (158–348 regions per donor). We tested for differences in average SNCA to IFN-γ genes correlations between the prenatal and postnatal samples. Consistent with the previous results, correlation drops from −0.025 in the prenatal dataset to −0.22 in the adult samples (p<0.01, Wilcoxon rank sum test). Again, empirical testing shows this is a specific result based on replacing IFN-γ genes with random sets of the same size (p<0.0001) and replacing SNCA with other genes (p = 0.029).

### Abnormal correlations between SNCA and IFN-γ genes in Parkinson’s

We next analyzed published datasets with postmortem gene expression profiles of substantia nigra to explore the correlations between SNCA and IFN-γ genes in diseased brains (Table 1). PD subjects and matched controls were assayed in three of these studies (Zhang et al., 2005; Moran et al., 2006; Lesnick et al., 2007). The fourth dataset profiled substantia nigra expression in 16 subjects with subclinical PD related Lewy body neuropathology and 17 matched controls [13]. For contrast we tested a fifth dataset that profiled expression in blood samples of 50 subjects at early PD stages (mean Hoehn and Yahr stage = 2.3) and 55 age matched controls [14].

**Figure 3:**
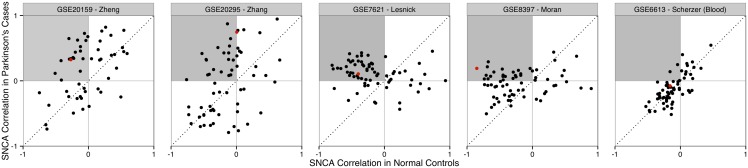
Scatterplot of SNCA co-expression with IFN-γ genes in five Parkinson’s studies. The first four studies assayed post-mortem substantia nigra samples with the final plot [14] showing co-expression results from blood. Genes switching from negative co-expression in normal controls to positive correlation in PD cases are highlighted in the shaded quadrant. The red point marks the IFNGR1 gene, which has the strongest change in co-expression in the substantia nigra datasets.

While these data do not allow tests of regional or spatial co-expression, we do observe co-expression differences across the substantia nigra samples. In all four substantia nigra datasets the mean correlation between SNCA and IFN-γ genes is negative for the healthy subjects, but positive or near zero in PD brains (Fig. 3, Table 1). The average increase in SNCA to IFN-γ correlations is 0.21 (p = 0.0041, Fisher’s trend of permutation tests). In contrast, mean correlation is unchanged in the blood of early stage PD and matched controls. This result is specific to SNCA, only 7 genes have larger co-expression increases with IFN-γ genes in all four substantia nigra datasets (SCN3B, RIT2, EFHD2, SCG2, PUS7, ANK1 and DOPEY1; p<0.002; n = 6748 genes). We note that Ras-Like without CAAX 2 (RIT2) has been recently associated with PD in two large genomic studies [27], [28] and Ankyrin 1, erythrocytic (ANK1) shows altered methylation and expression in Alzheimer’s disease [29], [30]. Specificity for IFN-γ genes was also high when compared to random gene sets of the same size (p<0.0002 for each dataset). Fig. 3 shows co-expression of individual genes, showing that on average 39% of IFN-γ genes switch from negative co-expression in normal controls to positive co-expression in Parkinson’s cases (p = 0.0003, Fisher’s trend of permutation tests, Table 1). Again, this result is specific to SNCA, with no other genes showing consistently more IFN-γ co-expression switches. Interferon gamma receptor 1 (IFNGR1) shows the largest SNCA co-expression difference between cases (mean rho = 0.34) and controls (mean rho = −0.38), suggesting a target link in the IFN-γ pathway (Fig. 3, S5 Table).

## Discussion

We examined co-expression patterns of SNCA in the brain, finding a strong negative correlation between SNCA and IFN-γ mediated signaling genes. This relationship amplifies with age in normal brain samples. We next examined correlation patterns of SNCA and IFN-γ in several transcriptomic datasets of PD and healthy brains and show a reversal of these correlations, from negative in healthy brains to positive in PD samples. Transcriptomic essays of PD samples demonstrate that the disease inflicts a global effect on patterns of gene expression [31]. However, the genes that are differentially expressed between PD and control samples lack agreement across studies, although some consistency is found in analyses of substantia nigra samples [32]. We show that the loss of negative correlation between SNCA and IFN-γ genes is significant, specific and robust.

Only a handful of genes scored show a higher specificity than SNCA, and a few of these are associated with Parkinson’s disease. Half of the six genes with larger increases in IFN-γ anti-correlations across age have been previously linked to Parkinson’s (UBE2E2 [26], PCMT1 [25] and HPRT1 [23], [24]). From the remaining three we note that Nucleosome assembly protein 1-like 2 (NAP1L2) and Mortality Factor 4 Like 2 (MORF4L2) function in histone acetylation [33], [34]. SNCA is also known to regulate histone acetylation [35], suggesting a broad mechanism that could downregulate the large set of IFN-γ genes. Less is known about the function of the seven genes with higher co-expression changes than SNCA in studies of Parkinson’s brain. Focused study in the context of IFN-γ and SNCA may help determine the role of these genes. Of these 7, genome-wide studies have associated RIT2 and ANK1 with Parkinson’s and Alzheimer’s disease respectively [27]–[30]. This shared property of differential IFN-γ co-expression suggests a possible grouping of cases based on disruptions of RIT2, ANK1 and SNCA.

Immunity is a major factor in Parkinons’s disease progression and immunomodulary therapies are being explored [36]. In agreement with our results, several human studies have linked IFN-γ to PD. Mount and colleagues report elevated blood plasma levels of IFN-γ in PD patients [37]. Genetically, a large number of the IFN-γ signaling genes are in the HLA histocompatibility region which harbors common variants that have been associated with PD [28], [38], [39]. Another PD risk gene, LRRK2, is suspected to be an IFN-γ target gene [40]. Epidemiological studies have found that cigarette smoking and coffee consumption confer reduced PD risk [41] and both reduce levels of IFN-γ production [42], [43]. More directly, use of nonsteroidal anti-inflammatory drugs are associated with decreased risk of PD [44].

At the cellular level, a fluorescence microscopy study of human derived glioblastomal cells treated with IFN-γ revealed a reduction of peripheral SNCA at low doses and aggregation after high concentration treatment of IFN-γ [45]. Although observed in malignant cells, this reduction in SNCA after low doses of IFN-γ parallels our negative co-expression observation in normal brain. The high dose response, like our findings in Parkinson’s cases, shows that the interaction between SNCA and IFN-γ is variable. Kim and colleagues have noted dual roles of SNCA: neuroprotection and neurotoxicity [46]. In addition, SNCA risk genotypes were found to have a dual and opposing associations with Parkinson’s symptom scores [47]. Our findings suggest IFN-γ signaling may provide these roles.

Experiments in mice have provided causal connections between IFN-γ and features of Parkinson’s that inform the correlations we found in postmortem brains samples. Specifically, the MHCII complex is required for microglia activation by SNCA expression [48]. Overexpression of IFN-γ causes neuronal loss primarily in the nigrostriatal tract and basal ganglia calcification [21]. Several PD-like features were reduced in IFN-γ deficient mice. *In vitro*, IFN-γ treatment causes microglia dependent death of dopamine neurons and mice treated with an IFN-γ neutralizing antibody had reduced rotenone-induced neuronal loss [37]. In Parkinsonian monkeys, IFN-γ levels correlated with motor impairment, microglia activation and damage to the substantia nigra [49]. Studies examining IFN-γ and SNCA interactions are lacking but *in vitro* studies suggest that microglia activation is modulated by SNCA. Microglia cultures from SNCA knockout mice show increased activation and cytokine secretion [50], [51]. These findings suggest interactions between SNCA and microglia via IFN-γ that are more direct than the response to SNCA aggregates. In agreement with others, our results suggest that therapeutic reduction of SNCA in PD may initiate unwanted changes in microglia phenotype [50] and Parkinson’s symptoms [47].

Our results combined with past observations in human and experiments in mouse and monkey provide more evidence for a role of IFN-γ in Parkinson’s. We specifically link SNCA to IFN-γ in the aging normal brain, suggesting immunomodulatory role of SNCA in later life. In diseased brains this negative co-expression with SNCA is lost with increased SNCA expression coinciding with increased IFN-γ signaling. This loss possibly causes IFN-γ microglia activation that leads to the dopaminergic cell loss seen in mouse and monkey. We suggest focus on discovering interactions between IFN-γ genes and SNCA in the normal brain to elucidate the molecular mechanisms involved in PD onset.

## Supporting Information

S1 Figure Differential co-expression between IFN-γ genes and SNCA across age in the exon array data.
(TIFF)

S1 Table Regional expression of SNCA in the Human Brain Atlas.
(XLS)

S2 Table Gene ontology groups enriched for positive SNCA co-expression. Each sheet in file represents results from a single Allen Human Brain Atlas donor.
(XLS)

S3 Table Gene ontology groups enriched for negative SNCA co-expression. Each sheet in file represents results from a single Allen Human Brain Atlas donor.
(XLS)

S4 Table Listing of the IFN-γ genes (GO:0060333).
(TXT)

S5 Table Co-expression differences between SNCA and each of the IFN-γ genes in the Parkinson’s microarray studies.
(XLS)
