# Supplementary figures and images for: Differential Co-Expression between α-Synuclein and IFN-γ Signaling Genes across Development and in Parkinson’s Disease
Source: PLoS One. 2014 Dec 10;9(12):e115029. doi: 10.1371/journal.pone.0115029 (PMC4262449; doi:10.1371/journal.pone.0115029)

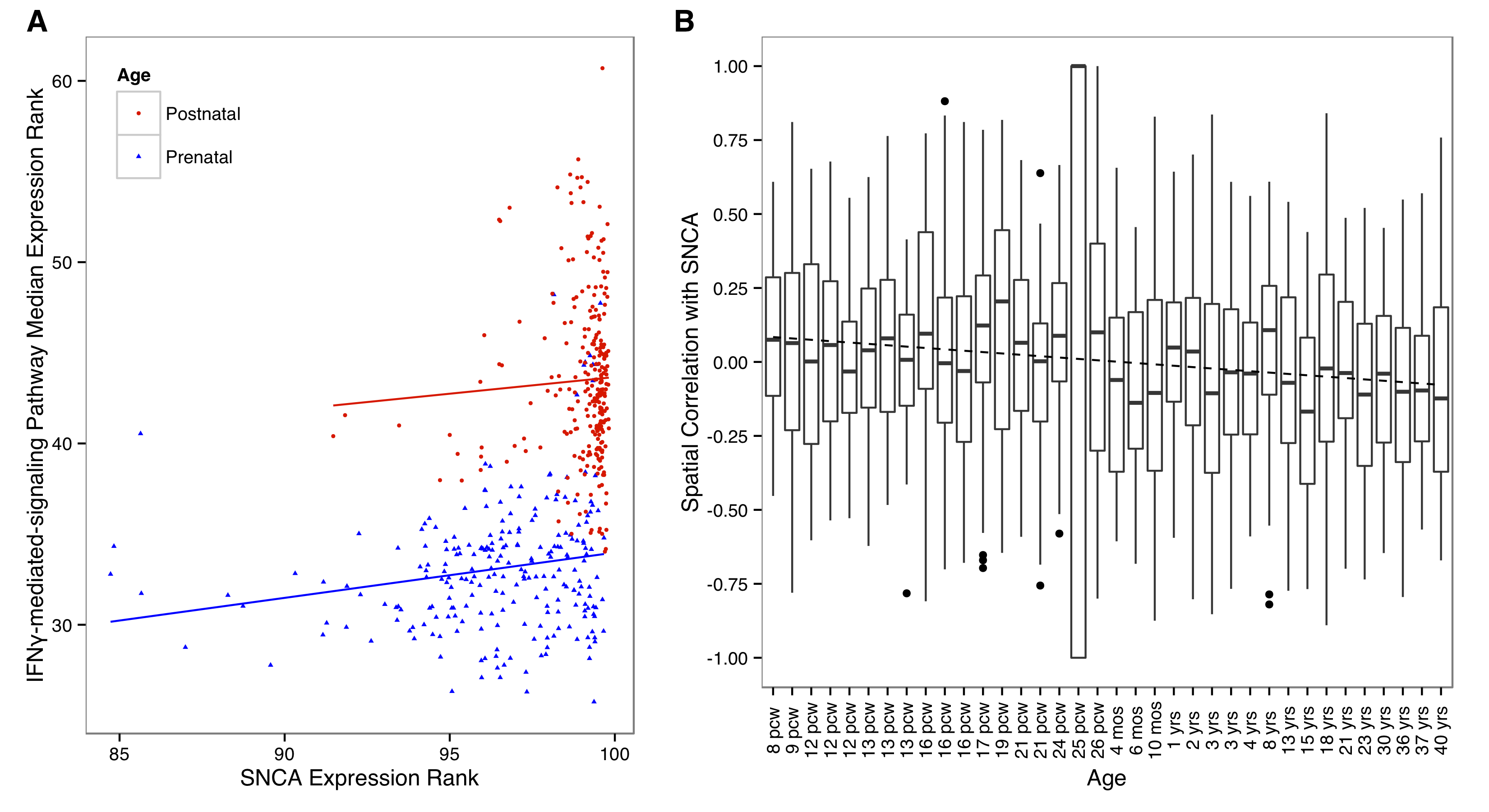

Supplement: S1 Figure — Differential co-expression between IFN-γ genes and SNCA across age in the exon array data. (TIFF) [file pone.0115029.s001.tiff]
